# The establishment of a prognostic scoring model based on the new tumor immune microenvironment classification in acute myeloid leukemia

**DOI:** 10.1186/s12916-021-02047-9

**Published:** 2021-08-05

**Authors:** Tiansheng Zeng, Longzhen Cui, Wenhui Huang, Yan Liu, Chaozeng Si, Tingting Qian, Cong Deng, Lin Fu

**Affiliations:** 1 Department of Hematology, The Second Affiliated Hospital, Guangzhou Medical University, Guangzhou, 510260 China; 2 Translational Medicine Center, State Key Laboratory of Respiratory Disease, The Second Affiliated Hospital, Guangzhou Medical University, Guangzhou, 510260 China; 3 Guangdong Provincial Education Department Key Laboratory of Nano-Immunoregulation Tumor Microenvironment, The Second Affiliated Hospital, Guangzhou Medical University, Guangzhou, 510260 China; 4 Translational Medicine Center, Huaihe Hospital of Henan University, Kaifeng, 475000 China; 5 Information Center, China-Japan Friendship Hospital, Beijing, 100029 China; 6 Department of Clinical Laboratory, The Second Affiliated Hospital of Guangzhou Medical University, Guangzhou, China; 7 Department of Hematology, Huaihe Hospital of Henan University, Kaifeng, 475000 China

**Keywords:** Acute myeloid leukemia, Tumor immune microenvironment classification, Prognostic model, Precision treatment

## Abstract

**Background:**

The high degree of heterogeneity brought great challenges to the diagnosis and treatment of acute myeloid leukemia (AML). Although several different AML prognostic scoring models have been proposed to assess the prognosis of patients, the accuracy still needs to be improved. As important components of the tumor microenvironment, immune cells played important roles in the physiological functions of tumors and had certain research value. Therefore, whether the tumor immune microenvironment (TIME) can be used to assess the prognosis of AML aroused our great interest.

**Methods:**

The patients’ gene expression profile from 7 GEO databases was normalized after removing the batch effect. TIME cell components were explored through Xcell tools and then hierarchically clustered to establish TIME classification. Subsequently, a prognostic model was established by Lasso-Cox. Multiple GEO databases and the Cancer Genome Atlas dataset were employed to validate the prognostic performance of the model. Receiver operating characteristic (ROC) and the concordance index (C-index) were utilized to assess the prognostic efficacy.

**Results:**

After analyzing the composition of TIME cells in AML, we found infiltration of ten types of cells with prognostic significance. Then using hierarchical clustering methods, we established a TIME classification system, which clustered all patients into three groups with distinct prognostic characteristics. Using the differential genes between the first and third groups in the TIME classification, we constructed a 121-gene prognostic model. The model successfully divided 1229 patients into the low and high groups which had obvious differences in prognosis. The high group with shorter overall survival had more patients older than 60 years and more poor-risk patients (both *P*< 0.001). Besides, the model can perform well in multiple datasets and could further stratify the cytogenetically normal AML patients and intermediate-risk AML population. Compared with the European Leukemia Net Risk Stratification System and other AML prognostic models, our model had the highest C-index and the largest AUC of the ROC curve, which demonstrated that our model had the best prognostic efficacy.

**Conclusion:**

A prognostic model for AML based on the TIME classification was constructed in our study, which may provide a new strategy for precision treatment in AML.

**Supplementary Information:**

The online version contains supplementary material available at 10.1186/s12916-021-02047-9.

## Background

Acute myeloid leukemia (AML) is a highly heterogeneous hematological malignancy characterized by clonal malignant proliferation of bone marrow progenitor cells [1]. Despite the continuous discovery of new therapeutic targets and drugs, the recurrence and mortality rates of AML are still high [2]. Accurate assessment of prognosis at the time of diagnosis is conducive to the treatment of the patients [3]. The European Leukemia Net (ELN) stratification system is the most widely used tool for stratifying the risk of AML patients, but the accuracy of this method needs to be improved [4]. There have been several prognostic models established with different foundations, such as microRNA, leukemia hematopoietic stem cells (LSC), and gene expression profiles [5–9]. However, these models still have some limitations, for example, the relatively small number of samples, complicated composition, and the inefficient validation in subtypes of AML. There is an urgent need to explore more optimized models.

The tumor microenvironment (TME) can play an immunosuppressive role in assisting the immune escape of tumor cells, which has attracted the attention of researchers [10]. Immune cells in TME mainly consist of natural killer (NKT) cells, macrophages, neutrophils, dendritic cells, suppressor cells derived from bone marrow, innate lymphoid cells, and T/B lymphocytes [11]. Great progress has been made in research on drugs that blocks the function of CTLA4 and PD-L1/PD1 in melanoma to release anti-tumor immunity [12, 13]. Unfortunately, through the immunosuppressive effect of TME that can promote tumor cell escape, those drugs showed limited efficacy for AML patients [14–16]. At present, the remodeling of the microenvironment and the restoration of an effective immune response still cannot be achieved, and relevant research is still very limited [17]. Furthermore, to the best of our knowledge, there is still a lack of an accurate prognostic model based on the immune microenvironment for AML. In this study, we used a large database to infer the tumor immune microenvironment of AML, established a tumor immune microenvironment (TIME) classification based on cell infiltration, and further constructed a prognostic model for AML patients, which may contribute to the diagnosis and treatment of AML.

## Methods

### Patients

We aimed to use all databases that were accessible and included overall survival (OS) data for the patients. At the time of this study, there was a total of eight AML cohorts containing gene expression data and corresponding clinical information. Seven of the cohorts were from the Gene Expression Omnibus (GEO) database (https://www.ncbi.nlm.nih.gov/geo/): GSE10358, GSE66525, GSE8970, GSE12417, GSE37642, GSE6891, and GSE71014. There were 1799 AML samples, of which 1229 samples had OS records. The Affymetrix microarray data sets of GSE10358, GSE12417, GSE37642, and GSE6891 were downloaded as CEL files and normalized by multi-chip averaging (R package affy, V1.60.0). The data of GSE71014, GSE66525, and GSE8970 were downloaded in the form of a normalized expression matrix. The eighth database came from the Cancer Genome Atlas (TCGA) database, which contained 173 AML patients with gene expression and prognosis data. Among these datasets, GSE12417 was only composed of cytogenetically normal (CN-) AML patients. The removed Batch Effect function of the R language limma package was used to remove batch effects [18], the expression matrices of the 7 GEO databases were merged, and then quantile normalization was performed through R package preprocessCore for model construction [19].

### Clustering of tumor immune microenvironment cells

The XCell tool (https://xcell.ucsf.edu) was used to analyze the expression matrix to infer the cellular components in the immune microenvironment of 1799 AML samples in the GEO datasets [20]. According to the median of each cell infiltration score, patients were divided into high and low groups, and the survival differences between the two groups were compared to evaluate the prognostic value of various cell infiltrations.

### Establishment of TIME classification

According to the infiltration scores of all cells with prognostic significance, all samples were hierarchically clustered based on Euclidean distance and Ward linkage to construct tumor immune microenvironment (TIME) classification.

### Construction and validation of AML prognostic model

The differential expression genes (DEGs) were analyzed by DEGseq. Those genes were considered as DEG by using thresholds for both the (adjusted) p-value and a fold-change (adj.P.Val < 0.05, FC > 1.5) [21]. Log-rank test and univariate COX regression analysis were used to screen differentially expressed genes with prognostic significance. DAVID was used (https://david.ncifcrf.gov/) for GO enrichment analysis, and the R software package was used for “GOPLOT” visualization [22]. Then the Cox-PH method based on Lasso was applied to establish the AML prognosis model. The risk index score of each patient was calculated, which was the sum of the expression of all genes in the model multiplied by their corresponding weighting coefficients. The median of the risk index scores in each group was identified as the cutoff value. Time-dependent receiver operating characteristic (ROC) curve analysis and Kaplan-Meier survival analysis were used to evaluate the predictive effect of risk index scores on the prognosis of AML patients (R package, survival ROC, v1.0.3).

To validate the predictive efficiency of the model, we firstly selected three GEO databases with a large number of patients: GSE10358, GSE37642, and GSE6891 for internal validation, and TCGA database for external validation; secondly, we chose GSE12417 to test the efficiency of our prognosis model in cytogenically normal AML (CN-AML) patients. At the same time, the GSE6891 and TCGA databases were selected to assess the prediction effects of the model in three different groups of AML patients, including good-risk, intermediate-risk (IR-), and poor-risk AML patients. All patients in the database were scored using the model, and the median score was used to divide the patients into high and low groups. Kaplan-Meier survival analysis was used to draw survival curves. The ELN system, LSC17, Wang and Yang models were collected, and the time/survival ROC curve and the concordance index (C-index) analysis were used to evaluate the prognostic performance.

### Statistical analyses

R version 3.6.3 was used for statistical analysis and mapping of data. The qualitative data was expressed by the number of cases and percentages. The Kaplan-Meier method log-rank test was used for survival analysis. *P* < 0.05 was considered as statistically significant.

## Results

### Clustering of immune cells in AML

The clinical information of AML patients from 7 GEO databases was shown in Table 1. Data information mainly came from 4 platforms, GPL10558 (n=104), GPL10532 (n=22), GPL570 (n=1055), and GPL96 (n=618). A total of 1799 patients were enrolled, of which 1299 patients had OS information. There were 479 patients in the group older than 60 years and 931 patients younger than 60 years. Regarding survival status, there were 449 patients alive, 884 patients died, and 457 patients with missing data.

**Table 1:** Summary of patient clinical information from 7 GEO databases

	Overall	GSE10358	GSE12417	GSE37642	GSE66525	GSE6891	GSE71014	GSE8970
n	1799	300	242	562	22	536	104	33
Platform (%)								
GPL10558	104 (5.8)	0 (0.0)	0 (0.0)	0 (0.0)	0 (0.0)	0 (0.0)	104 (100.0)	0 (0.0)
GPL11532	22 (1.2)	0 (0.0)	0 (0.0)	0 (0.0)	22 (100.0)	0 (0.0)	0 (0.0)	0 (0.0)
GPL570	1055 (58.6)	300 (100.0)	79 (32.6)	140 (24.9)	0 (0.0)	536 (100.0)	0 (0.0)	0 (0.0)
GPL96	618 (34.4)	0 (0.0)	163 (67.4)	422 (75.1)	0 (0.0)	0 (0.0)	0 (0.0)	33 (100.0)
Age group, year/n (%)								
< 60	931 (66.0)	53 (58.2)	119 (49.2)	297 (52.8)	14 (63.6)	448 (97.4)	0 (0.0)	0 (0.0)
≥ 60	479 (34.0)	38 (41.8)	123 (50.8)	265 (47.2)	8 (36.4)	12 (2.6)	0 (0.0)	33 (100.0)
WBC group, × 10^9^ L^−1^/n (%)								
< 10	567 (80.1)	4 (4.4)	0 (0.0)	562 (100.0)	0 (0.0)	0 (0.0)	0 (0.0)	1 (3.0)
≥ 10	141 (19.9)	87 (95.6)	0 (0.0)	0 (0.0)	22 (100.0)	0 (0.0)	0 (0.0)	32 (97.0)
PB blasts/n (%)								
< 40	50 (44.2)	48 (52.7)	0 (0.0)	0 (0.0)	2 (9.1)	0 (0.0)	0 (0.0)	0 (0.0)
≥ 40	63 (55.8)	43 (47.3)	0 (0.0)	0 (0.0)	20 (90.9)	0 (0.0)	0 (0.0)	0 (0.0)
BM blasts/n (%)								
< 50	1638 (97.9)	265 (88.3)	242 (100.0)	562 (100.0)	0 (0.0)	536 (100.0)	0 (0.0)	33 (100.0)
≥ 50	35 (2.1)	35 (11.7)	0 (0.0)	0 (0.0)	0 (0.0)	0 (0.0)	0 (0.0)	0 (0.0)
OS status/n (%)								
Alive	449 (25.1)	46 (15.3)	92 (38.0)	147 (26.6)	0 (0.0)	128 (23.9)	36 (34.6)	0 (0.0)
Dead	884 (49.4)	45 (15.0)	150 (62.0)	406 (73.4)	0 (0.0)	215 (40.1)	68 (65.4)	0 (0.0)
NA	457 (25.5)	209 (69.7)	0 (0.0)	0 (0.0)	22 (100.0)	193 (36.0)	0 (0.0)	33 (100.0)

Abbreviations: *WBC*, white blood cell; *BM*, bone marrow; *PB*, peripheral blood; *OS*, overall survival

**Fig. 1:**
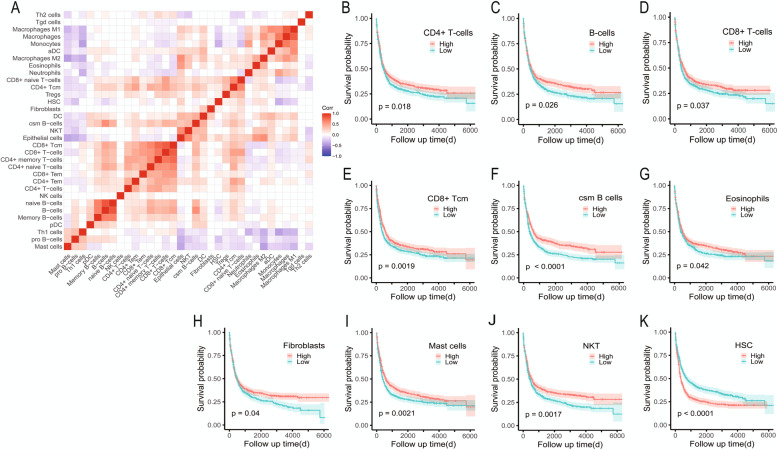
Cellular composition of AML immune microenvironment. **A** Correlation analysis of 33 different types of immune infiltrating cells. Red means positive correlation, blue means negative correlation, and blank means no significant correlation. **B**–**K**. Immune cells with prognostic significance in the tumor microenvironment. HSC, hematopoietic stem cells

To analyze the TIME of AML, we normalized the obtained GEO data after removing the batch effect and used the XCell tool to simulate and infer it by silico analysis. We found that there were 33 cell components in the AML immune microenvironment. Correlation matrix analysis showed that there were mainly B cell groups, T cell groups, and other cell groups (Fig. 1A). In order to evaluate the prognostic value of the infiltration level of these cells, all patients were divided into a high infiltration group and a low infiltration group according to the median cell infiltration score of each type, and the survival differences between the two groups were compared. The results of survival analysis showed that the high infiltration group of CD4^+^/CD8^+^ T cells, B cells, CD8^+^ central memory T cells, Class-switch memory B cells, eosinophils, fibroblasts, mast cells, and NKT cells were all conducive to survival (Fig. 1B–J; all *P* < 0.05); on the contrary, the group with high hematopoietic stem cell (HSC) infiltration had inferior OS (Fig. 1K; *P* < 0.0001).

### Establishment of AML immune microenvironment classification

**Fig. 2:**
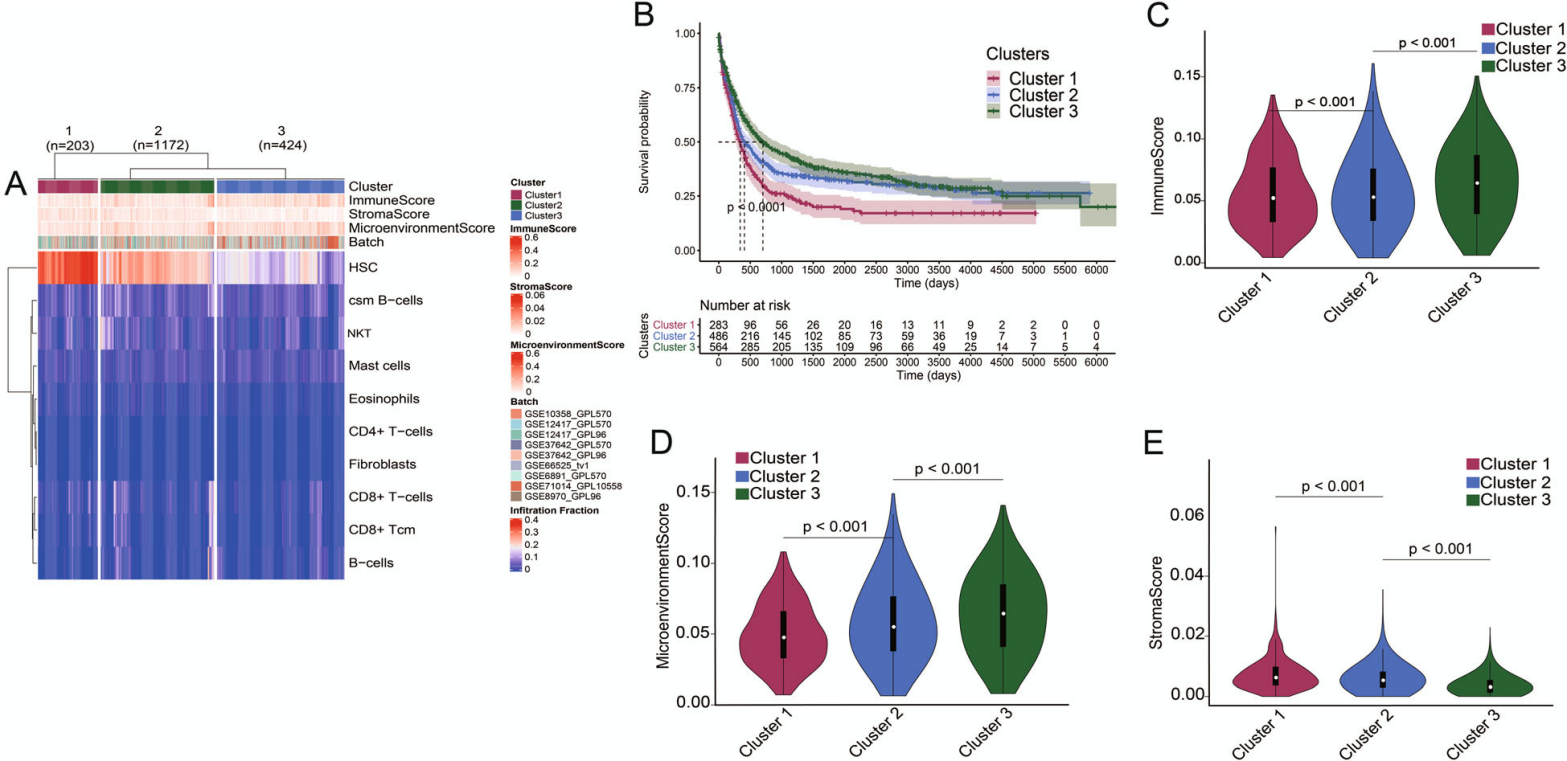
Establishment of the TIME classification of AML patients. **A** According to the information of 10 types of immune cells that had an impact on the prognosis, 1799 AML patients were clustered into three different groups, n (Cluster 1) = 203, n (Cluster 2) = 1172, and n (Cluster 3) = 424. **B** There were significant differences in the survival time of AML patients in three groups (*P* < 0.001). **C** There are significant differences in immune score of AML patients in three groups (*P* < 0.001). **D** There are significant differences in microenvironment score of AML patients in three groups (*P* < 0.001). **E** There are significant differences in stroma score among AML patients in three groups (*P* < 0.001). TIME, the tumor immune microenvironment

Based on the obtained AML immune microenvironmental cell information, we used infiltration levels of 10 types of cells with prognostic significance for hierarchically clustered AML patients to establish a TIME classification. All the patients were divided into three groups based on the TIME classification. There was a significant difference in survival between the three groups. Cluster 1 had the shortest survival time and Cluster 3 had the longest survival time (Fig. 2A, B). The score characteristics showed that Cluster 1 had the lowest immune score and microenvironment score, and the stroma score of Cluster 1 was the highest; Cluster 3 had the highest immune score and microenvironment score, and the stroma score of Cluster 3 was the lowest; the scores of the Cluster 2 were in the median (Fig. 2C–E).

### Construction of AML prognostic model based on TIME classification

**Fig. 3:**
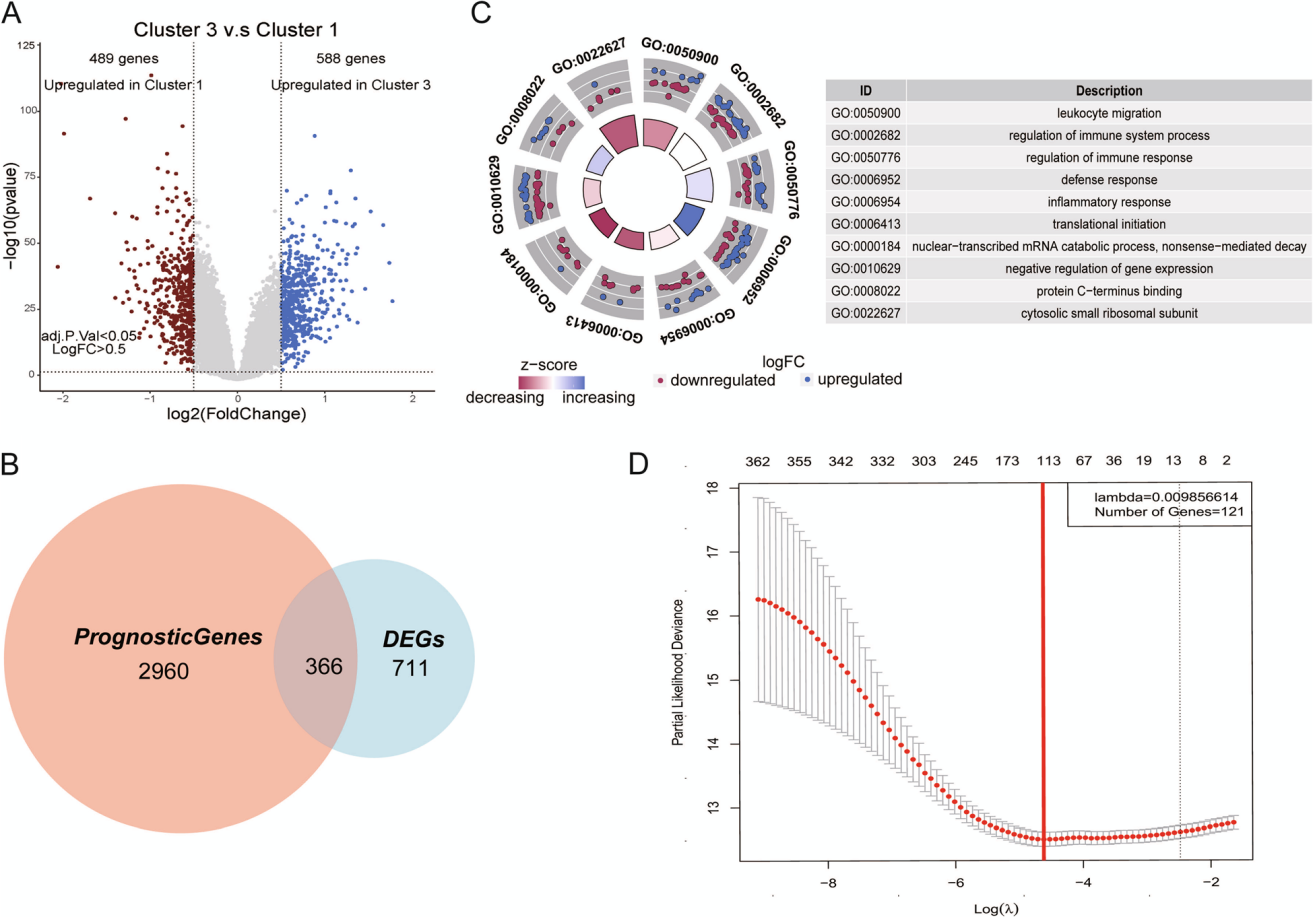
Construction of a prognostic model based on the TIME classification. **A** Volcano map to explore the differentially expressed genes between Cluster 1 and Cluster 3 groups. There were 489 highly expressed genes in Cluster 1 (red), and 588 highly expressed genes in Cluster 3 (blue). **B** Analysis of differentially expressed genes related to prognosis. The large circle represented 3326 genes that had an impact on the prognosis of AML, the small circle represented 1077 genes that were differentially expressed in Cluster 1 and Cluster 3, and the middle cross was 366 differentially expressed genes related to the prognosis. **C** Using 366 differentially expressed genes for GO annotation, it was found that differentially expressed genes were mainly enriched in the following pathways: leukocyte migration, regulation of immune system process, regulation of immune response, defense response, inflammatory response, translational initiation, nuclear-transcribed mRNA catabolic process, nonsense-mediated decay, negative regulation of gene expression, protein C-terminus binding, and cytosolic small ribosomal subunit. **D** Lasso regression analysis was used to construct a prognostic model containing 121 genes. TIME, the tumor immune microenvironment

To establish a prognostic model, we analyzed the DEGs in Cluster 1 and Cluster 3 which demonstrated the largest differences. Compared with Cluster 3, Cluster 1 had 489 upregulated genes and 588 downregulated genes. Among these 1077 DEGs, 366 genes had prognostic significance (Fig. 3A, B). GO analysis showed that these 366 genes were mainly involved in the regulation of the immune system, immune response, defense response, leukocyte migration, inflammatory response, and so on (Fig. 3C). LASSO-Cox was used to identify the genes which were most relevant to prognosis among the 366 DEGs with prognostic significance. The coefficient of each gene was calculated and a proportional hazard model containing 121 genes was established (Fig. 3D, Additional file 1: Table S1).

### Validation of AML prognostic model

**Fig. 4:**
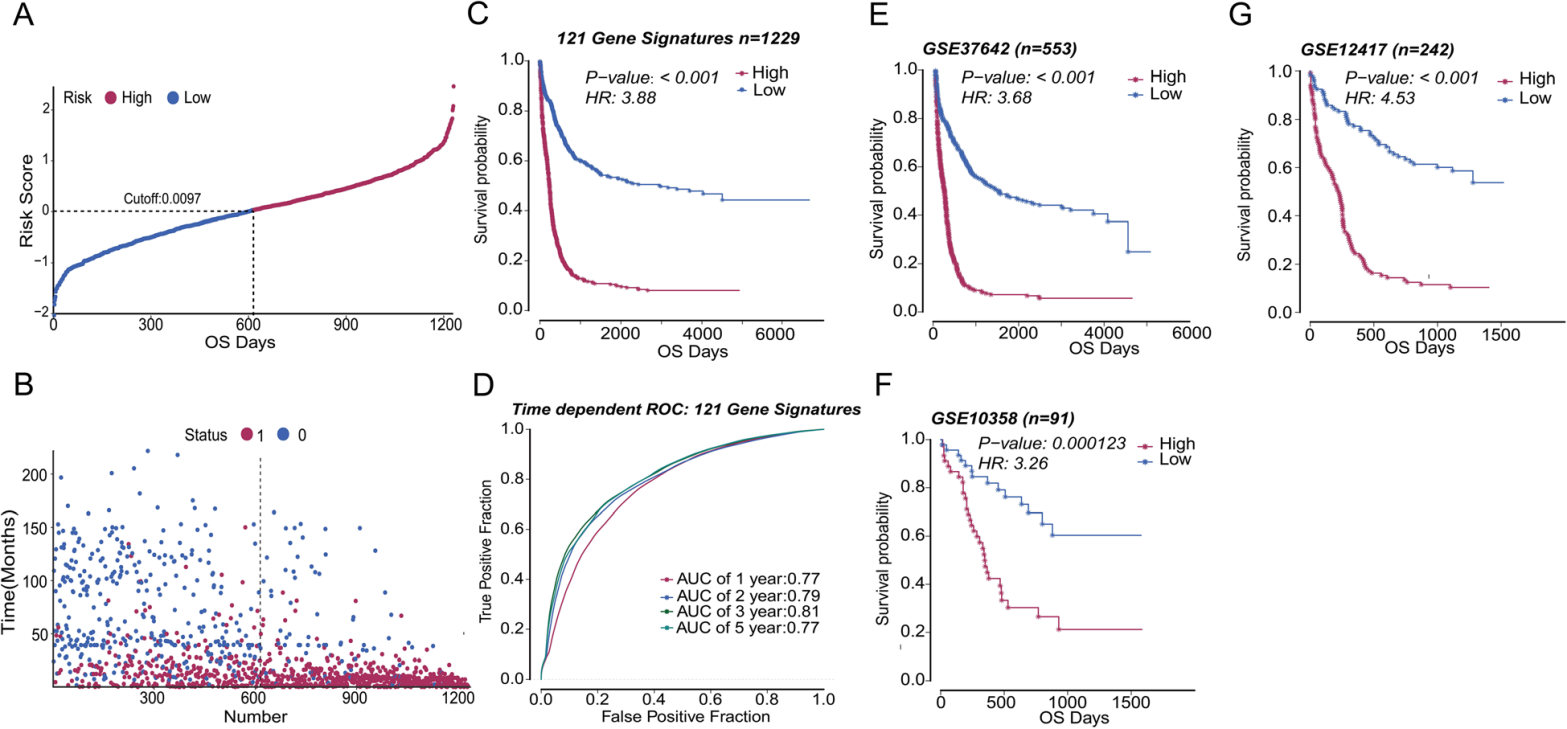
Validation of the prognostic model. **A** According to the median score of the prognostic model, patients were divided into high score group and low score group (Cutoff = 0.0097). **B** The scatter plot showed that more patients survived in the low score group (red represents death, blue represents survival). **C** Among all AML patients with OS data, AML patients in the high group had worse prognostic survival than those in the low group (n = 1229, *P* < 0.001). **D** The 1-, 2-, 3-, and 5-year AUC of AML patients obtained by the prognostic model were 0.77, 0.79, 0.81, and 0.77, respectively. **E** In GSE37642, the prognostic survival of AML patients in the higher group was shorter (n = 553, *P* < 0.001). **F** In GSE10358, AML patients with high score had a worse prognosis than patients with low score (n = 91, *P* < 0.001). **G** In GSE12417, which was all CN-AML, patients in the high group had worse prognostic survival than those in the low group (n = 242, *P* < 0.001). OS, overall survival; CN-AML, cytogenetically normal AML; AUC, area under the curve

After establishing the prognostic model, we verified the model with diversity AML cohorts. For 1229 AML patients, the calculated cutoff value was 0.0097, which equally divided the patients into the high and low groups (Fig. 4A). The higher the score, the shorter the patients’ survival time and the higher proportion of deaths (Fig. 4B). Subsequently, we used Kaplan-Meier survival analysis to compare the prognosis of two groups. Compared with the low score group, the survival status of the high score group was worse (Fig. 4C, *P* < 0.001). The clinical characteristics of patients in the two groups showed that the high score group had more old patients (age ≥ 60), fewer good- and intermediate-risk patients, and more poor-risk patients (Table 2, all *P* < 0.001). And the area under the curve (AUC) of 1, 2, 3, and 5 years were 0.77, 0.79, 0.81, and 0.77, respectively (Fig. 4D), indicating that our scoring model had high accuracy.

**Table 2:** Comparison of 1229 patients’ clinical characteristics in two groups

Characteristics	Total	High (n=614)	Low (n=615)	*P*
Age group/n (%)				< 0.001^§^
< 60 years	750 (61.0)	308 (50.2)	442 (71.9)	
≥ 60 years	428 (34.8)	276 (44.9)	152 (24.7)	
NA	51 (4.2)	30 (4.9)	21 (3.4)	
WBC/n (%)				0.798^§^
< 10 %	29 (2.4)	12 (2.0)	17 (2.8)	
≥ 10 %	62 (5.0)	29 (4.7)	33 (5.3)	
NA	1138 (92.6)	573 (93.3)	565 (91.9)	
PB blasts/n (%)				0.635^§^
< 40 %	48 (3.9)	20 (3.3)	28 (4.5)	
≥40 %	43 (3.5)	21 (3.4)	22 (3.6)	
NA	1138 (92.6)	573 (93.3)	565 (91.9)	
BM blasts /n (%)				0.462^§^
< 50 %	18 (1.5)	10 (1.6)	8 (1.3)	
≥ 50 %	73 (5.9)	31 (5.1)	42 (6.8)	
NA	1138 (92.6)	573 (93.3)	565 (91.9)	
Risk/n (%)				< 0.001^§^
Good	55 (4.5)	9 (1.5)	46 (7.5)	
Intermediate	175 (14.2)	67 (10.9)	108 (17.6)	
Poor	53 (4.3)	32 (5.2)	21 (3.4)	
NA	946 (77.0)	506 (82.4)	440 (71.5)	
OS/n (%)				< 0.001^§^
Alive	413 (33.6)	78 (12.7)	335 (54.5)	
Dead	816 (66.4)	536 (87.3)	280 (45.5)	

Abbreviations: *WBC*, white blood cell; *BM*, bone marrow; *PB*, peripheral blood; *NA*, not applicable

^§^Chi-square test

After analyzing the prediction efficiency across all the patients, we used independent GEO databases for validation. After processing the data in GSE34642 and GSE10358 with the same method, there was a significant difference in prognosis between the high score group and the low score group, and the high score was a poor prognostic factor (Fig. 4E, F). In a cohort with the same subtype of AML patients, such as GSE12417, which was all comprised of CN-AML patients, the model successfully divided the patients into high and low score groups with significant prognosis differences. The survival time of patients in the high score group was shorter, which was consistent with the results of other databases (Fig. 4G).

**Fig. 5:**
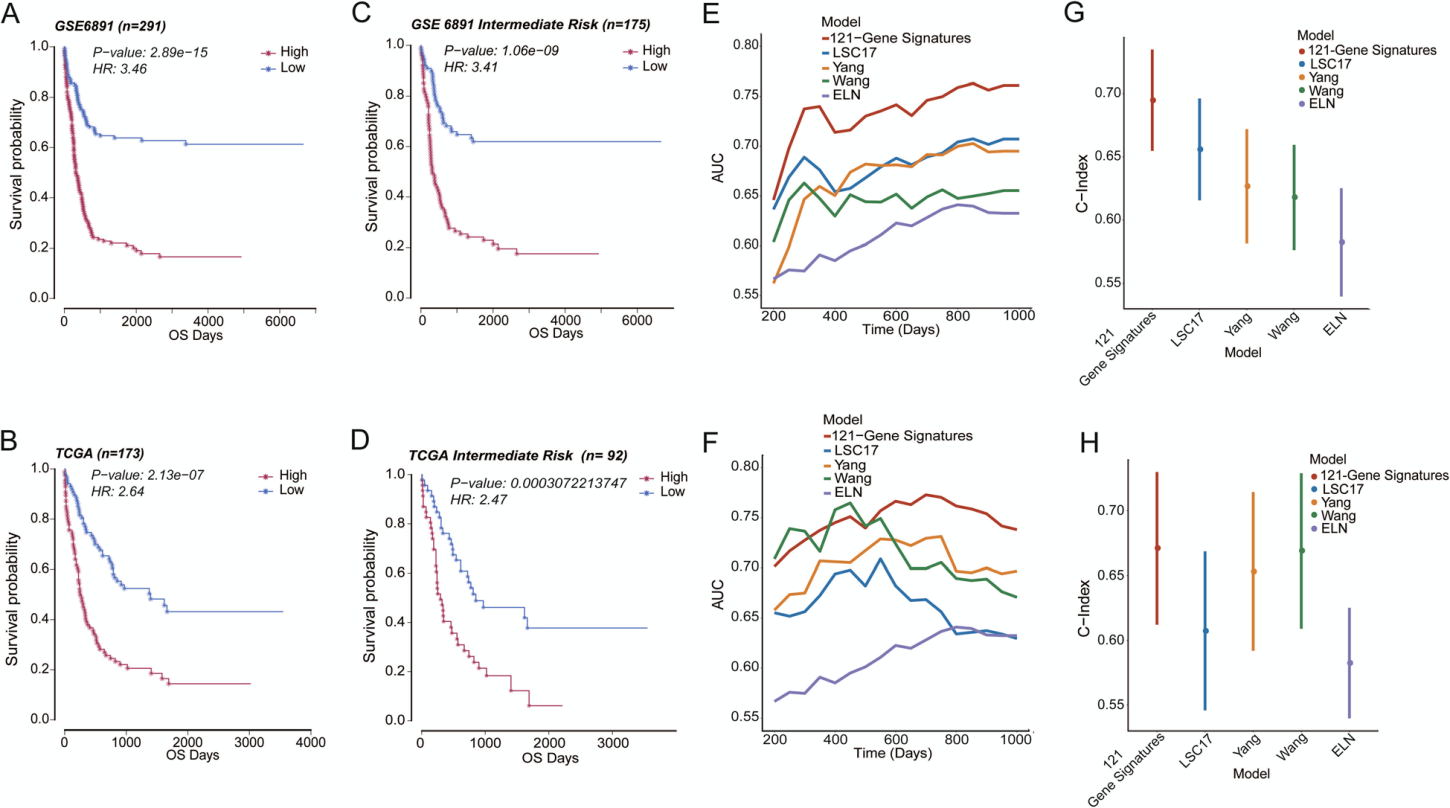
The comparison of the prognostic model with the ELN system and other models. **A**, **B** In GSE6891 (n=291) and the TCGA database (n=173), AML patients in the high-score group had worse prognostic survival than those in the low-score group (both *P* < 0.001). **C**, **D** The model divided the IR-AML patients into two groups with significant differences in survival (both *P* < 0.001) in GSE6891 (n=175) and the TCGA database (n = 92). In GSE6891 and the TCGA database, **E**, **F** compared with LSC17, Yang, Wang’ models, and ELN system, the 121-gene prognostic model had the highest AUC value of survival ROC. **G**, **H** The 121-gene prognostic model had a higher C-Index value than the LSC17, Yang, Wang’ models, and the ELN system. IR-AML, intermediate-risk AML; AUC, area under the curve; ROC, receiver operating characteristic; C-index, concordance index

Moreover, GSE6891 and the TCGA database were selected to test the model’s predictive performance in a different stratification. Our model performed well in these two databases, which divided patients into two groups with significant differences in prognosis, and the OS of patients in the high score group was shorter (Fig. 5A, B; both *P* < 0.001). Similar results were also found in the intermediate risk AML (IR-AML) patients in two cohorts (Fig. 5C, D; both *P* < 0.001). For patients in the good-risk group, there was no significant difference in both cohorts (Additional file 1: Fig. S1A, B). Finally, the model divided the poor-risk patients of GSE6891 into two groups with different prognoses, and the group with higher scores had inferior OS (Additional file 1: Fig. S1C, *P*=0.0094). However, the same result was not found in the TCGA database, most likely due to the small number of poor-risk patients (Additional file 1: Fig. S1D).

In recent years, some new AML prognostic models have been proposed. We selected the latest three models to compare their prediction effects with our model: Wang’s model, LSC-17, and Yang’s model [5–7]. Wang et al. established a model based on the gene expression profiling (RNA sequencing), which demonstrated the best predictive performance compared with previous studies. Therefore, we also compared the models published after Wang. LSC-17 was established on the basis of AML hematopoietic stem cells, and Yang et al. constructed a model using the gene expression profiling. In addition, we also compared our model with the classic ELN risk stratification system. Multivariate survival analysis found that in the GSE6891 and the TCGA database, our 121-gene prognostic model was the only independent prognostic factor for AML patients (Table 3; both *P* < 0.05). In GSE6891 and the TCGA database, our prognostic model demonstrated the largest AUC of the survival ROC curve and highest C-Index among the five prognostic models, indicating that our prognostic model was more reliable (Fig. 5E–H). At the same time, we also calculated the AUC of the time ROC curve and statistically analyzed the results of the four models. The final results showed that in GSE6891, our model had the highest AUC. The comparison of AUC between our model and LSC17 displayed the significant differences at all times (Additional file 1: Fig. S2A, all *P* < 0.05). Comparing our model with the Wang and Yang models, there were significant differences in AUC from the 600th day (Additional file 1: Fig. S2A, all *P* < 0.05). In the TCGA database, although our model demonstrated the largest AUC from the 500th day, there was only a statistical difference between our model and LSC17 on the 1000th day (Additional file 1: Fig. S2B, *P*=0.042). The possible reason for this result was the small number of patients in the TCGA database.

**Table 3:** Multivariable overall survival analysis in the TCGA cohort and GSE6891

Variable	TCGA			GSE6891		
	Coef	HR (95%CI)	*P*	Coef	HR (95%CI)	*P*
121-Gene-Signatures	0.390	1.477 (1.004–2.172)	0.048	1.252	3.499 (2.043–5.992)	< 0.001
LSC17	− 0.989	0.372 (0.035–3.913)	0.410	− 0.009	0.991 (0.702–1.398)	0.959
Yang	0.955	2.599 (0.150–45.074)	0.512	− 0.777	0.460 (0.069–3.046)	0.421
Wang	0.322	1.381 (0.588–3.244)	0.459	0.146	1.157 (0.625–2.145)	0.642
ELN risk stratification						
Good	NA	NA	NA	− 0.317	0.729 (0.298–1.779)	0.487
Intermediate	0.677	1.969 (0.822–4.714)	0.128	− 0.420	0.657 (0.310–1.395)	0.274
Poor	0.742	2.099 (0.786–5.606)	0.139	− 0.616	0.540 (0.244–1.194)	0.128
Age (≥ 60 vs. < 60 years)	0.505	1.657 (0.981–2.799)	0.059	0.013	1.013 (0.999–1.027)	0.063
WBC (≥10 × 10^9^ vs. < 10 × 10^9^/L)	0.254	1.289 (0.764–2.175)	0.341	NA	NA	NA
BM blasts (≥ 50 vs. < 50%)	0.631	1.879 (0.853–4.140)	0.118	NA	NA	NA
PB blasts (≥ 40 vs. < 40%)	0.108	1.114 (0.633–1.962)	0.708	NA	NA	NA
*FLT3_ITD* (positive vs. negative)	0.003	1.003 (0.404–2.491)	0.994	− 0.220	0.803 (0.536–1.203)	0.287
*NPM1* (mutated vs. wild)	− 0.242	0.785 (0.389–1.586)	0.500	0.478	1.613 (1.057–2.461)	0.027
*DNMT3A* (mutated vs. wild)	0.296	1.345 (0.788–2.295)	0.277	NA	NA	NA
*RUNX1* (mutated vs. wild)	− 0.302	0.739 (0.307–1.783)	0.501	NA	NA	NA
*CEBPA* (mutated vs. wild)	NA	NA	NA	0.137	1.147 (0.448–2.936)	0.776
*IDH1* (mutated vs. wild)	NA	NA	NA	− 0.389	0.678 (0.389–1.180)	0.169
*IDH2* (mutated vs. wild)	NA	NA	NA	0.384	1.468 (0.817–2.637)	0.199
*NRAS* (mutated vs. wild)	NA	NA	NA	0.035	1.036 (0.596–1.801)	0.901
*KRAS* (mutated vs. wild)	NA	NA	NA	− 0.526	0.591 (0.179–1.955)	0.389

Abbreviations: *WBC*, white blood cell; *BM*, bone marrow; *PB*, peripheral blood

## Discussion

In this study, we used 7 AML GEO databases to infer the cellular composition of the immune microenvironment and construct the TIME classification based on infiltration characteristics of 10 types of immune cells. In TIME classification, patients were divided into three groups with significant differences in survival. Cluster 1 had the worst prognosis, and Cluster 3 had the best prognosis. The scoring characteristics of the three groups showed that the status of immunity and the number of HSCs had opposite prognostic effects on AML patients. The patients with the stronger immune function had the better prognosis, while the patients with a greater number of HSCs demonstrated the worse prognosis. The reasons for this were also well understood. The patients with more powerful immunity have stronger abilities to kill AML tumor cells, which tend to have the longer survival time. The HSCs in this study included normal HSCs and LSCs. LSCs mainly exist in patients and can drive disease recurrence [23]. High infiltration of HSCs in TIME was a poor prognostic factor. In addition to evaluating the prognosis, the TIME classification could also be used to construct a prognostic scoring model.

Our prognostic scoring model was validated internally and externally in multiple databases and showed excellent prognostic performance. Multivariate analysis showed that our model was the only independent risk factor compared with other models. At the same time, our model displayed the largest AUC and highest C-index. Collectively, our model had better prediction accuracy. Multi-time points AUC in our model were significantly higher than other models in GSE6891 cohorts; however, in the TCGA cohort, only 1000th day AUC of our model showed significantly higher than other models. This may be due to the small number and the high heterogeneity in clinical characteristics of patients in the TCGA cohort.

The prognostic prediction of AML has always been a relatively complex issue. Our model cannot further stratify good-risk AML patients which may be due to the relatively small number of patients. In the poor-risk patient group, there were similar survival curves in the two databases. Owing to the insufficient number of patients, there was no statistical difference in the TCGA database. Because high-risk patients were easily identified and their treatment strategies were mature, we mainly focused on the IR-AML patient group. The current ELN risk stratification system sometimes misclassified the IR-AML patients. Moreover, the intermediate-risk group is the largest subgroup with marked clinical heterogeneity. Our AML prognostic model based on TIME classification successfully reclassified the IR-AML patients in the GSE6891 and the TCGA database, enabling the more accurate treatment for these patients. In addition, our model can also distinguish the poor prognosis group from CN-AML patients without cytogenetic abnormalities, providing new possibilities for personalized treatment.

In conclusion, we used 7 AML cohorts with a large sample size to build a prognostic model. AML patients in different ages and patients with different cytogenetic abnormalities were enrolled in our study. The predictive effect of the model has been successfully validated in multiple databases, indicating that the model had an excellent prognostic performance. However, the prognostic model had some limitations. For example, it was derived from the retrospective research and was still not clinically applicable at present. In the future, these shortcomings may be overcome through prospective experiments and the invention of novel multiplex Polymerase Chain Reaction kits. The prognostic model may also have other effects on clinical implementations, such as using these genes to find potential therapeutic targets and drugs, which may provide new ideas for the diagnosis and treatment of AML.

## Conclusion

We aggregated multiple database information to establish the TIME classification of AML patients. A new prognostic model was constructed based on classification, and the predictive effect of the model had been validated in different AML databases. It can further group CN-AML and IR-AML, and its predictive efficiency was better than the ELN system and other published new models. This model provided a new method for predicting the prognosis of AML patients and discovered new ways for clinical diagnosis and treatment.

## Supplementary Information

**Additional file 1.** Table S1; Figures S1-S2. Table S1 - Data information of 121 genes involved in the AML prediction model. FigS1 - The application effect of the AML prognostic model in the good-risk patient group and the poor-risk patient group. FigS2 - The comparison of the AUC value of the time ROC curve between the 121-gene signatures model, previous published models and the ELN system.

## Data Availability

All GEO databases in this article can be downloaded on the GEO website (https://www.ncbi.nlm.nih.gov/geo/) [24–30]. The TCGA dataset comes from http://www.tcga.org/ [31].

## Abbreviations

AML: Acute myeloid leukemia
GEO: Gene Expression Omnibus
TCGA: The Cancer Genome Atlas
LSC: Leukemia hematopoietic stem cells
TME: The tumor microenvironment
TIME: The tumor immune microenvironment
CN-AML: Cytogenetically normal AML
IR-AML: Intermediate-risk AML
HSC: Hematopoietic stem cells
WBC: White blood cell
BM: Bone marrow
PB: Peripheral blood
OS: Overall survival
AUC: The area under the curve
ROC: Receiver operating characteristic
C-index: Concordance index
DEG: Differential expression gene

## References

[1] Juliusson G, Lazarevic V, Hörstedt A-S, Hagberg O, Höglund M, Group SALR (2012). Acute myeloid leukemia in the real world: why population-based registries are needed. Blood..

[2] Buckley SA, Kirtane K, Walter RB, Lee SJ, Lyman GH (2018). Patient-reported outcomes in acute myeloid leukemia: where are we now?. Blood Rev.

[3] Weinberg OK, Sohani AR, Bhargava P, Nardi V (2017). Diagnostic work-up of acute myeloid leukemia. Am J Hematol.

[4] Komanduri KV, Levine RL (2016). Diagnosis and therapy of acute myeloid leukemia in the era of molecular risk stratification. Annu Rev Med.

[5] Wang M, Lindberg J, Klevebring D, Nilsson C, Lehmann S, Grönberg H, Rantalainen M (2018). Development and validation of a novel RNA sequencing-based prognostic score for acute myeloid leukemia. J Natl Cancer Inst.

[6] Yang Z, Shang J, Li N, Liang Z, Tang T, Tian G (2020). Development and validation of a 10-gene prognostic signature for acute myeloid leukaemia. J Cell Mol Med.

[7] Ng SWK, Mitchell A, Kennedy JA, Chen WC, McLeod J, Ibrahimova N (2016). A 17-gene stemness score for rapid determination of risk in acute leukaemia. Nature..

[8] Chuang M-K, Chiu Y-C, Chou W-C, Hou H-A, Chuang EY, Tien H-F (2015). A 3-microRNA scoring system for prognostication in de novo acute myeloid leukemia patients. Leukemia..

[9] Li Z, Herold T, He C, Valk PJM, Chen P, Jurinovic V (2013). Identification of a 24-gene prognostic signature that improves the European LeukemiaNet risk classification of acute myeloid leukemia: an international collaborative study. J Clin Oncol.

[10] Hanahan D, Coussens LM (2012). Accessories to the crime: functions of cells recruited to the tumor microenvironment. Cancer Cell.

[11] Hinshaw DC, Shevde LA (2019). The tumor microenvironment innately modulates cancer progression. Cancer Res.

[12] Arasanz H, Gato-Cañas M, Zuazo M, Ibañez-Vea M, Breckpot K, Kochan G (2017). PD1 signal transduction pathways in T cells. Oncotarget.

[13] Stephen Hodi F, O'Day SJ, McDermott DF, Weber RW, Sosman JA, Haanen JB (2010). Improved survival with ipilimumab in patients with metastatic melanoma. N Engl J Med.

[14] Coles SJ, Gilmour MN, Reid R, Knapper S, Burnett AK, Man S, Tonks A, Darley RL (2015). The immunosuppressive ligands PD-L1 and CD200 are linked in AML T-cell immunosuppression: identification of a new immunotherapeutic synapse. Leukemia..

[15] Austin R, Smyth MJ, Lane SW (2016). Harnessing the immune system in acute myeloid leukaemia. Crit Rev Oncol Hematol.

[16] Isidori A, Salvestrini V, Ciciarello M, Loscocco F, Visani G, Parisi S, Lecciso M, Ocadlikova D, Rossi L, Gabucci E, Clissa C, Curti A (2014). The role of the immunosuppressive microenvironment in acute myeloid leukemia development and treatment. Expert Rev Hematol.

[17] Yu Y-R, Ho P-C (2019). Sculpting tumor microenvironment with immune system: from immunometabolism to immunoediting. Clin Exp Immunol.

[18] Ritchie ME, Phipson B, Di Wu YH, Law CW, Shi W (2015). limma powers differential expression analyses for RNA-sequencing and microarray studies. Nucleic Acids Res.

[19] Zhao Y, Wong L, Goh WWB (2020). How to do quantile normalization correctly for gene expression data analyses. Sci Rep.

[20] Aran D, Hu Z, Butte AJ (2017). xCell: digitally portraying the tissue cellular heterogeneity landscape. Genome Biol.

[21] Wang L, Feng Z, Wang X, Wang X, Zhang X (2010). DEGseq: an R package for identifying differentially expressed genes from RNA-seq data. Bioinformatics..

[22] Walter W, Sánchez-Cabo F, Ricote M (2015). GOplot: an R package for visually combining expression data with functional analysis. Bioinformatics..

[23] Mattes K, Vellenga E, Schepers H (2019). Differential redox-regulation and mitochondrial dynamics in normal and leukemic hematopoietic stem cells: A potential window for leukemia therapy. Crit Rev Oncol Hematol.

[24] Ley TJ, Tomasson MH, Payton JE. Discovery and validation of expression data for the Genomics of Acute Myeloid Leukemia Program at Washington University. GEO. https://www.ncbi.nlm.nih.gov/geo/query/acc.cgi?acc=GSE10358. (2008).

[25] Verhaak RG, Wouters BJ, Erpelinck CA, Abbas S, Beverlo B, Lugthart S, et al. Acute myeloid leukemia samples of samples =< 60yrs on HG-U133 plus 2. GEO. http://odin.mdacc.tmc.edu/~rverhaak/. 2008.

[26] Wieser R, Hackl H. A gene expression profile associated with relapse of cytogenetically normal acute myeloid leukemia is enriched for leukemia stem cell genes. GEO. https://www.ncbi.nlm.nih.gov/geo/query/acc.cgi?acc=GSE66525. (2015). 10.3109/10428194.2014.944523 PMC4695919 25030037

[27] Raponi M. A two-gene classifier for predicting response to the farnesyltransferase inhibitor tipifarnib in acute myeloid leukemia. GEO. https://www.ncbi.nlm.nih.gov/geo/query/acc.cgi?acc=GSE8970. (2007). 10.1182/blood-2007-09-112730 18160667

[28] Metzeler KH, Hummel M, Mansmann U, Hiddemann W, Bohlander SK, Buske C. Prognostic gene signature for normal karyotype AML. GEO. https://www.ncbi.nlm.nih.gov/geo/query/acc.cgi?acc=GSE12417. (2008). 10.4137/bbi.s1018 PMC2735947 19812786

[29] Herold T, Jurinovic V, Metzeler KH, Sauerland MC, Pasalic Z, Dufour A, et al. Prognostic gene signature for AML. GEO. https://www.ncbi.nlm.nih.gov/geo/query/acc.cgi?acc=GSE37642. 2013.

[30] Chou W, Tien H. An mRNA expression signature for prognostication in de novo acute myeloid leukemia patients with normal karyotype. geo. https://www.ncbi.nlm.nih.gov/geo/query/acc.cgi?acc=GSE71014. (2017). 10.18632/oncotarget.5390 PMC4770759 26517675

[31] Ley TJ, Miller C, Ding L, Raphael BJ, Mungall AJ, Network; CGAR (2013). Genomic and epigenomic landscapes of adult de novo acute myeloid leukemia. N Engl J Med.

