# Supplementary material for: The establishment of a prognostic scoring model based on the new tumor immune microenvironment classification in acute myeloid leukemia
Source: BMC Med. 2021 Aug 5;19:176. doi: 10.1186/s12916-021-02047-9 (PMC8340489; doi:10.1186/s12916-021-02047-9)
Supplement: Supplementary file 1 — Additional file 1. Table S1; Figures S1-S2. Table S1 - Data information of 121 genes involved in the AML prediction model. FigS1 - The application effect of the AML prognostic model in the good-risk patient group and the poor-risk patient group. FigS2 - The comparison of the AUC value of the time ROC curve between the 121-gene signatures model, previous published models and the ELN system. [file 12916_2021_2047_MOESM1_ESM.docx]

**Table S1. Data information of 121 genes that made up the AML prediction model**

| No. | Gene | coef | HR | upper95 | lower95 | *P* |  | No. | Gene | coef | HR | upper95 | lower95 | *P* |
| --- | --- | --- | --- | --- | --- | --- | --- | --- | --- | --- | --- | --- | --- | --- |
| 1 | ABCC1 | -0.04536 | 1.18 | 1.345 | 1.032 | 0.0154 |  | 62 | HSPD1 | 0.056464 | 1.22 | 1.398 | 1.07 | 0.00312 |
| 2 | ACP6 | 0.053694 | 1.41 | 1.609 | 1.231 | 4.90E-07 |  | 63 | HTR1F | -0.0673 | 1.22 | 1.391 | 1.068 | 0.00332 |
| 3 | ACSL4 | -0.09119 | 0.813 | 0.931 | 0.71 | 0.00267 |  | 64 | IDI1 | 0.046594 | 1.21 | 1.385 | 1.06 | 0.00482 |
| 4 | ADA2 | -0.06985 | 0.778 | 0.893 | 0.678 | 0.000349 |  | 65 | IL17RA | 0.00695 | 0.861 | 0.984 | 0.754 | 0.0272 |
| 5 | ADGRG1 | 0.053812 | 1.72 | 1.981 | 1.5 | 7.33E-15 |  | 66 | IQGAP1 | 0.032438 | 0.85 | 0.971 | 0.744 | 0.0168 |
| 6 | AGA | -0.01081 | 0.82 | 0.936 | 0.719 | 0.00328 |  | 67 | ITGA6 | -0.0236 | 1.14 | 1.306 | 1.003 | 0.0445 |
| 7 | AGFG1 | 0.026436 | 0.807 | 0.924 | 0.705 | 0.00183 |  | 68 | KCTD3 | 0.058009 | 1.17 | 1.338 | 1.027 | 0.0184 |
| 8 | ANKRD28 | 0.028102 | 1.28 | 1.459 | 1.12 | 0.000272 |  | 69 | LAPTM4B | 0.057557 | 1.55 | 1.775 | 1.359 | 7.98E-11 |
| 9 | APBB1IP | -0.01176 | 0.796 | 0.909 | 0.697 | 0.000725 |  | 70 | LGALS2 | -0.10883 | 0.872 | 0.995 | 0.764 | 0.0419 |
| 10 | APOBR | -0.00646 | 0.743 | 0.853 | 0.647 | 2.29E-05 |  | 71 | LILRA2 | 0.00116 | 0.861 | 0.982 | 0.754 | 0.0258 |
| 11 | ARPC5L | 0.119308 | 1.36 | 1.554 | 1.188 | 7.38E-06 |  | 72 | LPAR4 | 0.029245 | 0.866 | 0.988 | 0.758 | 0.0321 |
| 12 | BAX | -0.0052 | 0.836 | 0.955 | 0.731 | 0.00829 |  | 73 | MAGED1 | -0.07027 | 1.21 | 1.384 | 1.063 | 0.00416 |
| 13 | BCAP29 | -0.11993 | 0.745 | 0.852 | 0.652 | 1.53E-05 |  | 74 | MFSD10 | -0.03457 | 0.842 | 0.961 | 0.738 | 0.0105 |
| 14 | BCAT1 | 0.007916 | 1.23 | 1.402 | 1.076 | 0.00224 |  | 75 | MN1 | -0.20074 | 1.21 | 1.379 | 1.058 | 0.00497 |
| 15 | BEX3 | 0.032732 | 1.52 | 1.753 | 1.326 | 2.56E-09 |  | 76 | MS4A6A | 0.177882 | 0.874 | 0.997 | 0.766 | 0.0452 |
| 16 | BPGM | 0.026197 | 0.862 | 0.983 | 0.755 | 0.027 |  | 77 | MYL4 | -0.26235 | 0.873 | 0.997 | 0.765 | 0.0446 |
| 17 | CBX4 | -0.06124 | 0.81 | 0.925 | 0.709 | 0.0018 |  | 78 | NBPF1 | -0.04536 | 1.17 | 1.341 | 1.028 | 0.0175 |
| 18 | CCNA1 | -0.03143 | 0.848 | 0.967 | 0.743 | 0.0141 |  | 79 | NIN | 0.092477 | 0.838 | 0.959 | 0.733 | 0.0102 |
| 19 | CCT4 | -0.21123 | 1.18 | 1.347 | 1.032 | 0.0153 |  | 80 | NRIP1 | -0.10027 | 1.27 | 1.452 | 1.115 | 0.000341 |
| 20 | CD34 | 0.044311 | 1.27 | 1.445 | 1.11 | 0.000439 |  | 81 | OPTN | 0.09672 | 1.16 | 1.322 | 1.014 | 0.0305 |
| 21 | CD58 | 0.023291 | 0.806 | 0.92 | 0.706 | 0.00136 |  | 82 | P2RX7 | -0.01966 | 0.843 | 0.963 | 0.738 | 0.0118 |
| 22 | CES1 | 0.037469 | 0.855 | 0.976 | 0.748 | 0.0206 |  | 83 | PDE12 | 0.02172 | 0.861 | 0.983 | 0.754 | 0.0271 |
| 23 | CFAP70 | -0.08777 | 1.2 | 1.381 | 1.046 | 0.0096 |  | 84 | PF4 | -0.00246 | 1.15 | 1.312 | 1.008 | 0.038 |
| 24 | CIAO1 | 0.062363 | 0.865 | 0.989 | 0.757 | 0.0332 |  | 85 | PGGT1B | -0.01232 | 0.81 | 0.926 | 0.709 | 0.00198 |
| 25 | CISD1 | 0.006665 | 1.15 | 1.311 | 1.006 | 0.0406 |  | 86 | PHLDA1 | 0.070917 | 1.18 | 1.343 | 1.028 | 0.0176 |
| 26 | CKLF | 0.025459 | 1.14 | 1.307 | 1.002 | 0.046 |  | 87 | PIK3C2B | 0.057878 | 1.3 | 1.486 | 1.141 | 8.58E-05 |
| 27 | CKM | 9.02E-05 | 0.804 | 0.918 | 0.704 | 0.00124 |  | 88 | PLK4 | 0.03737 | 0.872 | 0.995 | 0.763 | 0.0425 |
| 28 | CLIC2 | 0.159003 | 1.38 | 1.582 | 1.211 | 1.67E-06 |  | 89 | PNMA1 | -0.01642 | 1.19 | 1.357 | 1.042 | 0.00994 |
| 29 | CPVL | 0.019446 | 1.21 | 1.386 | 1.063 | 0.00418 |  | 90 | PNRC2 | -0.00562 | 1.2 | 1.368 | 1.049 | 0.00778 |
| 30 | CRIPT | -0.00872 | 0.84 | 0.96 | 0.735 | 0.0106 |  | 91 | PRKCH | -0.17435 | 1.15 | 1.31 | 1.005 | 0.0411 |
| 31 | CTSB | 0.075752 | 0.847 | 0.967 | 0.741 | 0.0137 |  | 92 | RAB3GAP2 | -0.15907 | 0.857 | 0.98 | 0.75 | 0.024 |
| 32 | CXCL2 | 0.054328 | 1.3 | 1.487 | 1.137 | 0.000123 |  | 93 | RAP1A | 0.029188 | 1.27 | 1.452 | 1.11 | 0.000481 |
| 33 | CXorf21 | 0.089499 | 0.829 | 0.95 | 0.724 | 0.007 |  | 94 | RBPMS | -0.07385 | 1.33 | 1.519 | 1.166 | 2.13E-05 |
| 34 | CYFIP2 | -0.02961 | 1.22 | 1.389 | 1.066 | 0.00356 |  | 95 | RCL1 | -0.04004 | 1.15 | 1.314 | 1.007 | 0.0389 |
| 35 | DEFB1 | 0.012026 | 0.791 | 0.903 | 0.693 | 0.000508 |  | 96 | REC8 | 0.015634 | 1.39 | 1.589 | 1.214 | 1.47E-06 |
| 36 | DNMT3B | 0.206895 | 1.4 | 1.595 | 1.224 | 6.47E-07 |  | 97 | RPS23 | -0.08356 | 1.19 | 1.362 | 1.041 | 0.0111 |
| 37 | DOCK1 | 0.105598 | 1.4 | 1.607 | 1.227 | 7.22E-07 |  | 98 | RUNX3 | 0.032893 | 1.25 | 1.428 | 1.095 | 0.000936 |
| 38 | DSG2 | 0.00505 | 1.3 | 1.482 | 1.135 | 0.00013 |  | 99 | SALL2 | -0.0074 | 1.14 | 1.304 | 1.001 | 0.0486 |
| 39 | EFCAB2 | -0.12283 | 0.794 | 0.907 | 0.695 | 0.000647 |  | 100 | SCRN1 | 0.028521 | 1.59 | 1.812 | 1.387 | 9.93E-12 |
| 40 | EHD2 | -0.00511 | 1.26 | 1.443 | 1.107 | 0.000499 |  | 101 | SELENOP | 0.007612 | 1.18 | 1.352 | 1.025 | 0.0205 |
| 41 | EIF3H | -0.15834 | 1.19 | 1.359 | 1.038 | 0.0125 |  | 102 | SLC2A6 | -0.01728 | 0.8 | 0.914 | 0.7 | 0.00101 |
| 42 | EZR | 0.045924 | 0.862 | 0.984 | 0.755 | 0.0275 |  | 103 | SLC38A1 | 0.035821 | 1.39 | 1.593 | 1.222 | 7.80E-07 |
| 43 | F2RL1 | 0.077141 | 1.64 | 1.871 | 1.429 | 4.68E-13 |  | 104 | SMIM10L1 | 0.063606 | 1.27 | 1.461 | 1.103 | 0.000842 |
| 44 | FAM124B | 0.085853 | 1.45 | 1.659 | 1.268 | 5.01E-08 |  | 105 | SNU13 | 0.090761 | 1.2 | 1.38 | 1.043 | 0.0107 |
| 45 | FAM30A | 0.0013 | 1.65 | 1.881 | 1.439 | 1.73E-13 |  | 106 | SOCS2 | 0.131483 | 1.61 | 1.84 | 1.408 | 2.00E-12 |
| 46 | FCGRT | 0.092483 | 0.856 | 0.977 | 0.75 | 0.0207 |  | 107 | ST8SIA4 | -0.04455 | 0.838 | 0.957 | 0.733 | 0.00882 |
| 47 | FECH | -0.13624 | 0.819 | 0.935 | 0.718 | 0.003 |  | 108 | SV2A | 0.071534 | 1.21 | 1.382 | 1.061 | 0.00442 |
| 48 | FGD2 | 0.045351 | 0.831 | 0.95 | 0.726 | 0.00664 |  | 109 | TCEAL9 | 3.66E-05 | 1.51 | 1.741 | 1.318 | 3.98E-09 |
| 49 | FHL1 | 0.013819 | 1.5 | 1.714 | 1.313 | 1.88E-09 |  | 110 | TCF4 | 0.014388 | 1.47 | 1.677 | 1.287 | 1.07E-08 |
| 50 | FSTL1 | 0.084853 | 1.2 | 1.372 | 1.053 | 0.00641 |  | 111 | TGOLN2 | -0.04739 | 0.874 | 0.998 | 0.766 | 0.0459 |
| 51 | FZD6 | 0.074265 | 1.41 | 1.615 | 1.237 | 3.05E-07 |  | 112 | TMCO3 | -0.1207 | 0.867 | 0.99 | 0.76 | 0.0343 |
| 52 | GABRB2 | 0.113903 | 1.3 | 1.491 | 1.137 | 0.000131 |  | 113 | TMEM176B | -0.02183 | 0.826 | 0.943 | 0.724 | 0.00454 |
| 53 | GNAI1 | 0.074098 | 1.2 | 1.373 | 1.055 | 0.00587 |  | 114 | TNFRSF21 | 0.003175 | 1.27 | 1.452 | 1.112 | 0.000429 |
| 54 | GTF2F2 | 0.075657 | 0.87 | 0.994 | 0.762 | 0.0397 |  | 115 | TPK1 | -0.00135 | 0.836 | 0.954 | 0.732 | 0.00778 |
| 55 | GYPC | -0.13728 | 0.849 | 0.968 | 0.744 | 0.0147 |  | 116 | TRAPPC11 | 0.078161 | 0.837 | 0.963 | 0.728 | 0.0125 |
| 56 | H1F0 | 0.121942 | 1.3 | 1.487 | 1.141 | 8.82E-05 |  | 117 | TTC28 | -0.00666 | 1.39 | 1.592 | 1.22 | 9.53E-07 |
| 57 | HAL | -0.01489 | 0.779 | 0.889 | 0.682 | 0.000208 |  | 118 | TUBGCP5 | 0.043584 | 0.842 | 0.963 | 0.736 | 0.012 |
| 58 | HDAC4 | -0.02931 | 0.763 | 0.871 | 0.669 | 5.97E-05 |  | 119 | VAMP8 | -0.05866 | 0.853 | 0.974 | 0.747 | 0.0186 |
| 59 | HDGFL3 | -0.04063 | 1.16 | 1.326 | 1.007 | 0.039 |  | 120 | ZEB2 | -0.04877 | 0.851 | 0.972 | 0.746 | 0.0172 |
| 60 | HOMER3 | -0.02963 | 0.782 | 0.893 | 0.685 | 0.000272 |  | 121 | ZNHIT1 | -0.04197 | 0.856 | 0.977 | 0.75 | 0.021 |
| 61 | HOPX | 0.157316 | 1.24 | 1.417 | 1.086 | 0.00147 |  |  |  |  |  |  |  |  |


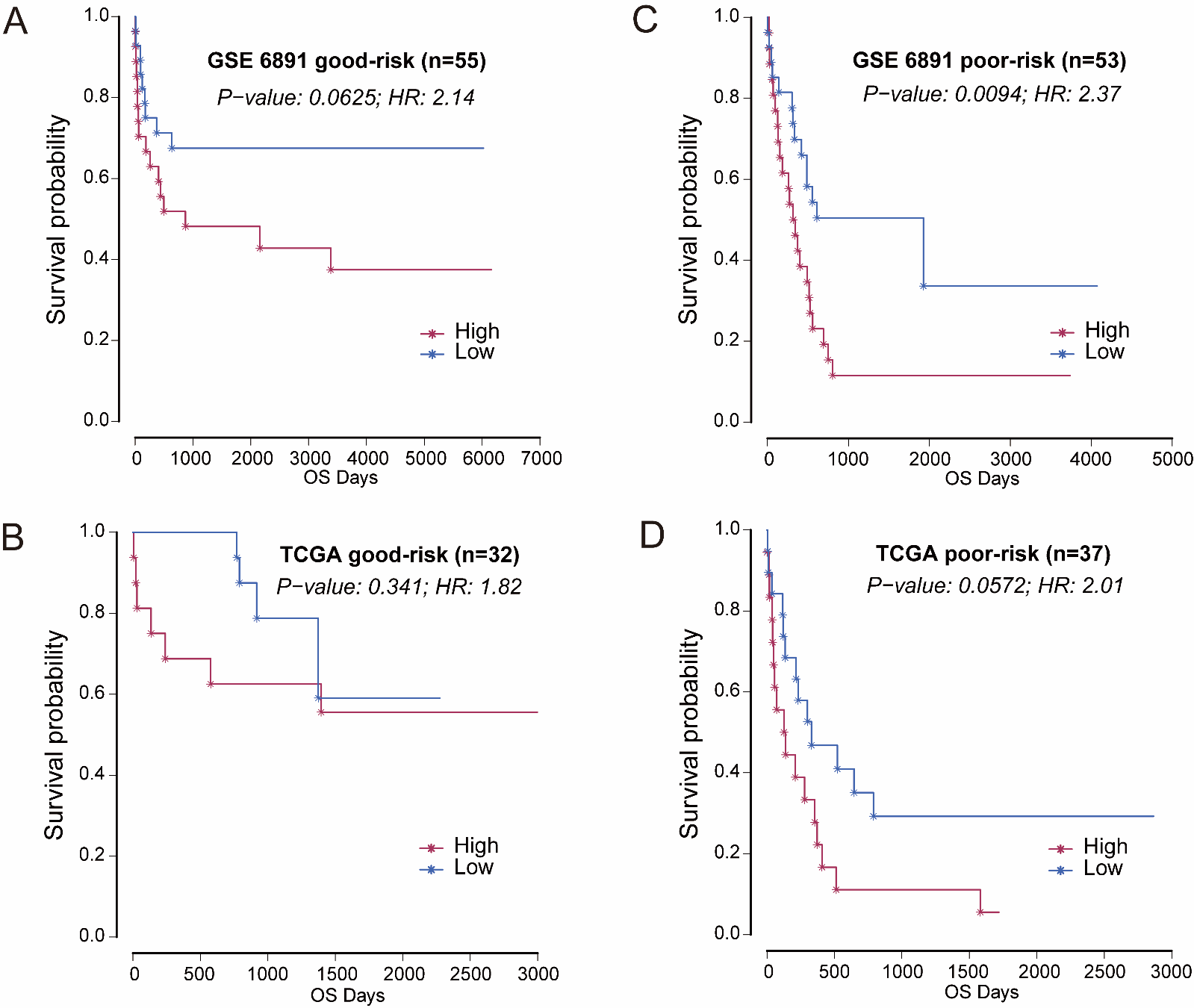


**Fig. S1.** The application effect of the AML prognostic model in the good-risk patient group and the poor-risk patient group. A. In GSE6891, the prognostic model did not successfully group good-risk patients (*P*=0.0625, n=55). B. In the TCGA database, the prognostic model did not successfully group good-risk patients (*P*=0.341, n=32). C. In GSE6891, the prognosis model divided poor-risk patients into two groups with significant differences in prognosis (*P*=0.0094, n=53). D. In the TCGA database, the prognostic model did not successfully group poor-risk patients (P=0.0572, n=37). TCGA: The Cancer Genome Atlas.


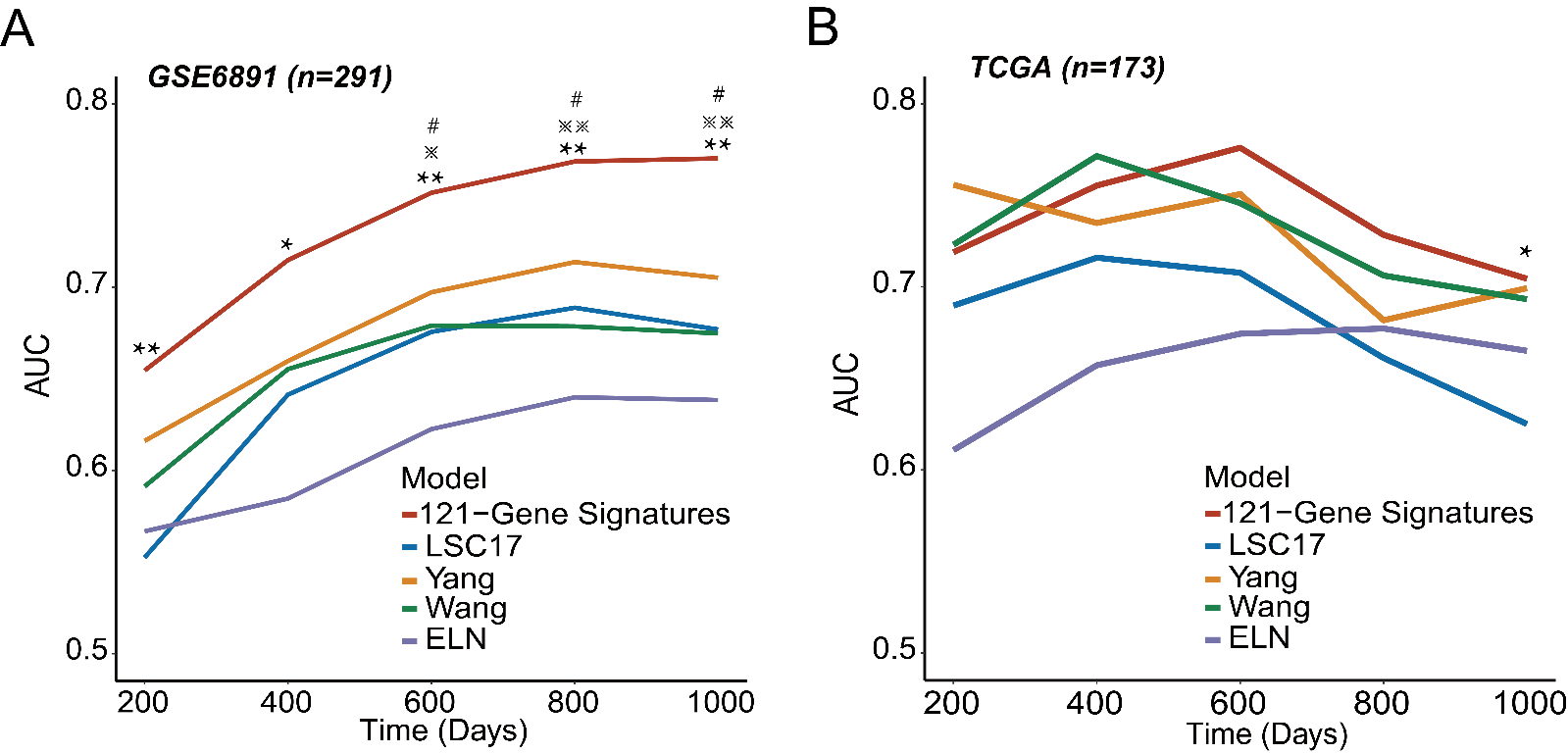


**Fig. S2**. The comparison results of the AUC value of the time ROC curve between the 121-gene signatures model, several other models and the ELN system. A. In GSE6891 cohort, the 121-gene signatures model had the highest AUC value of the time ROC curve compared with reference model (LSC17 model, Yang’s model, Wang’s model, and the ELN system). And most of the differences were statistically significant. B. In the TCGA cohort, the 121-gene signatures model had the highest AUC value of the time ROC curve. On the 1000th day, the AUC value between the 121-gene signatures model and the LSC17 model had a significant difference. * represented the comparison between the 121-gene signatures model and the LSC17 model; ＃represented the comparison between the 121-gene signatures model and the Yang’s model; ※ represented the comparison between the 121-gene signatures model and the Wang’s model; */＃/※ meant *P*<0.05; **/※※ meant *P*<0.001. AUC: Area Under the Curve; ROC: Receiver Operating Characteristic; ELN: European LeukemiaNet; TCGA: The Cancer Genome Atlas.

.
